# Barriers to Diabetes Care in Developing Countries: A Systematic Review of Patient, Provider, and Healthcare Challenges

**DOI:** 10.7759/cureus.108023

**Published:** 2026-04-30

**Authors:** Miriam A Okorie, Collins C Okeke, Onyinye E. Ebiliekwe, Praise C Dumbili, Otutochukwu O Ike-Obioha, Desmond E Orie, Omosimisola O Alli, Chinecherem C Ezema, Oluwatosin A Shaba, Miliete Berhe, Sylvahelen Okorienta, Nneka E Olisakwe, Chinemerem Ogbonna, Ephraim U Okeke

**Affiliations:** 1 Emergency Medicine, East Lancashire Hospitals NHS Trust, Blackburn, GBR; 2 Internal Medicine, University of Port Harcourt Teaching Hospital, Port Harcourt, NGA; 3 Internal Medicine, Nnamdi Azikiwe University Teaching Hospital, Nnewi, NGA; 4 Radiology, Delta State University, Abraka, Abraka, NGA; 5 Medicine and Surgery, University of Port Harcourt, Port Harcourt, NGA; 6 Internal Medicine, Delta State University Teaching Hospital, Oghara, Oghara, NGA; 7 General Practice, Lagos State University College of Medicine, Lagos, NGA; 8 General Practice, Afe Babalola University College of Medical and Health Science, Port Harcourt, NGA; 9 Medicine, Ayder Comprehensive Specialized Hospital, Mekelle University, Mekelle, ETH; 10 Public Health, Liberty University, Lynchburg, USA; 11 Integrative Medicine, Nnamdi Azikiwe University Teaching Hospital, Nnewi, NGA; 12 Internal Medicine, Enugu State University of Science and Technology Teaching Hospital, Enugu, NGA

**Keywords:** barrier to healthcare, developing countries, diabetes care, diabetes management, diabetes mellitus, medication adherence

## Abstract

Diabetes mellitus (DM) is a major global health problem with greater impact in developing countries. Despite increasing awareness, effective diabetes management in these settings is hindered by multiple barriers. This systematic review aims to identify and synthesize the barriers to effective diabetes care in developing countries.

A comprehensive literature search of PubMed and Google Scholar was conducted from inception to February 16, 2026, and 1,127 articles were synthesized; 14 articles met the predefined inclusion criteria. We included original articles published in peer-reviewed journals and individuals of any gender and age who were managed for diabetes mellitus in any developing country and reported any barriers, challenges, or limitations to diabetes care. The Joanna Briggs Institute (JBI) risk-of-bias critical appraisal tool for cross-sectional studies was used.

Barriers to diabetes care in developing countries are complex, with financial limitations and health system inefficiencies playing a central role. Addressing these challenges requires integrated strategies that target healthcare financing, workforce capacity, and patient education to improve diabetes outcomes.

## Introduction and background

Diabetes mellitus (DM) is a chronic metabolic disorder characterized by impaired pancreatic insulin secretion or ineffective insulin utilization, resulting in persistent hyperglycemia. It is diagnosed by fasting plasma glucose of >126mg/dl or a random plasma glucose level of >200 mg/dl [1,2]. The burden of type 2 diabetes in developing countries has been on the increase. Some ethnic groups (e.g., South Asians and Africans) tend to develop diabetes a decade earlier compared to the white population and have an accelerated conversion from prediabetes to diabetes. Also, the burden of its complications, both macro- and microvascular, is substantial but varies across the population [3].

Recent estimations from the International Diabetes Federation (IDF) Diabetes Atlas (11th edition) highlighted diabetes as one of the fastest-growing global health challenges of the twenty-first century. In 2024, approximately 589 million adults aged 20-79 years were living with diabetes worldwide, with projections indicating a rise to 853 million by 2050 [4]. In addition, over 9.5 million individuals were living with type 1 diabetes, including 1.9 million children and adolescents under the age of 20 years. A substantial proportion of the global population remains at high risk, with an estimated 635 million individuals having impaired glucose tolerance and 488 million having impaired fasting glucose in 2024. Diabetes also contributes significantly to mortality and economic burden, accounting for over 3.4 million deaths among adults aged 20-79 years and generating healthcare expenditures exceeding one trillion USD globally [4].

Several risk factors contribute to the increasing prevalence of diabetes, including family history, advancing age, obesity, sedentary lifestyle, and comorbid conditions such as non-alcoholic fatty disease [5]. In developing countries, these risk factors may be exacerbated by environmental and socioeconomic conditions such as limited access to healthy food options, lack of physical activity, and lack of healthcare infrastructure.

The rising prevalence of diabetes in developing countries, coupled with constrained healthcare resources, contributes to a substantial clinical and socioeconomic burden. This includes increased mortality, a high prevalence of microvascular and macrovascular complications, reduced quality of life, increased healthcare costs and productivity losses, all of which negatively impact individuals, families, and broader communities [6].

Despite the growing burden, effective diabetes care in developing countries is often hindered by multiple interconnected barriers. These include patient-related factors, provider-related challenges, and healthcare system challenges. These barriers collectively contribute to delayed diagnosis, suboptimal glycemic control, and increased risk of complications. Therefore, this systematic review aims to identify and synthesize the barriers to effective diabetes care in developing countries.

## Review

Methods

This systematic review was conducted in accordance with Preferred Reporting Items for systematic reviews and meta-analysis (PRISMA 2020). The study protocol was registered with PROSPERO (CRD420261334944).

Eligibility

We included original articles published in a peer-reviewed journal and included any individual of any gender and age who has been managed for diabetes mellitus in any developing country and reported any barriers, challenges, or limitations to diabetes care.

Exclusion Criteria

We excluded those that didn't discuss barriers/limitations/difficulties in the management of diabetes mellitus in a developing country. We also excluded non-English articles, reports, comments, abstracts, surveys, case reports, case series, editorials, systematic reviews, and meta-analyses.

Search Strategy and Data Screening

A systematic search was conducted from database inception to February 16, 2026, on PubMed and Google Scholar (displayed only 50 pages) databases with the following search phrases across the database: (barrier) AND (Diabetes) AND (management) AND (developing country), "barrier" "Diabetes" "management" "developing country". More details are shown in Appendix A.

The search results were imported into the Rayyan referencing manager (Rayyan Systems Inc., Cambridge, MA, USA) [7] and were used to screen our articles, where duplicate, title, and abstract screening were carried out by three independent co-authors. Following abstract screening, the eligible articles were subjected to full-text screening using the pre-defined eligibility criteria. Disagreements were resolved among the authors, and another co-author was consulted when necessary.

Data Extraction and Quality Assessment

Data extraction from the eligible articles was done by four co-authors independently into a Google spreadsheet. The extracted baseline variables include: author's name, country, study year, sample size, gender, mean age, and barrier to diabetes care. Included studies were assessed using the Joanna Briggs Institute (JBI) critical appraisal checklist [8]. The cross-sectional study demonstrated high methodological quality; the included studies defined the inclusion criteria, the study populations and settings in detail, and used a valid method for measuring exposure and outcomes. Potential cofounding factors were identified, with appropriate strategies reported to address them. The JBI critical appraisal tool does not have standardized grading to report articles with a high or low risk of bias. Despite these limitations, the overall quality of the included studies was considered high, with a generally low risk of bias. JBI graded yes(Y), no(N), and maybe as shown in Appendix B.

Results

**Figure 1:**
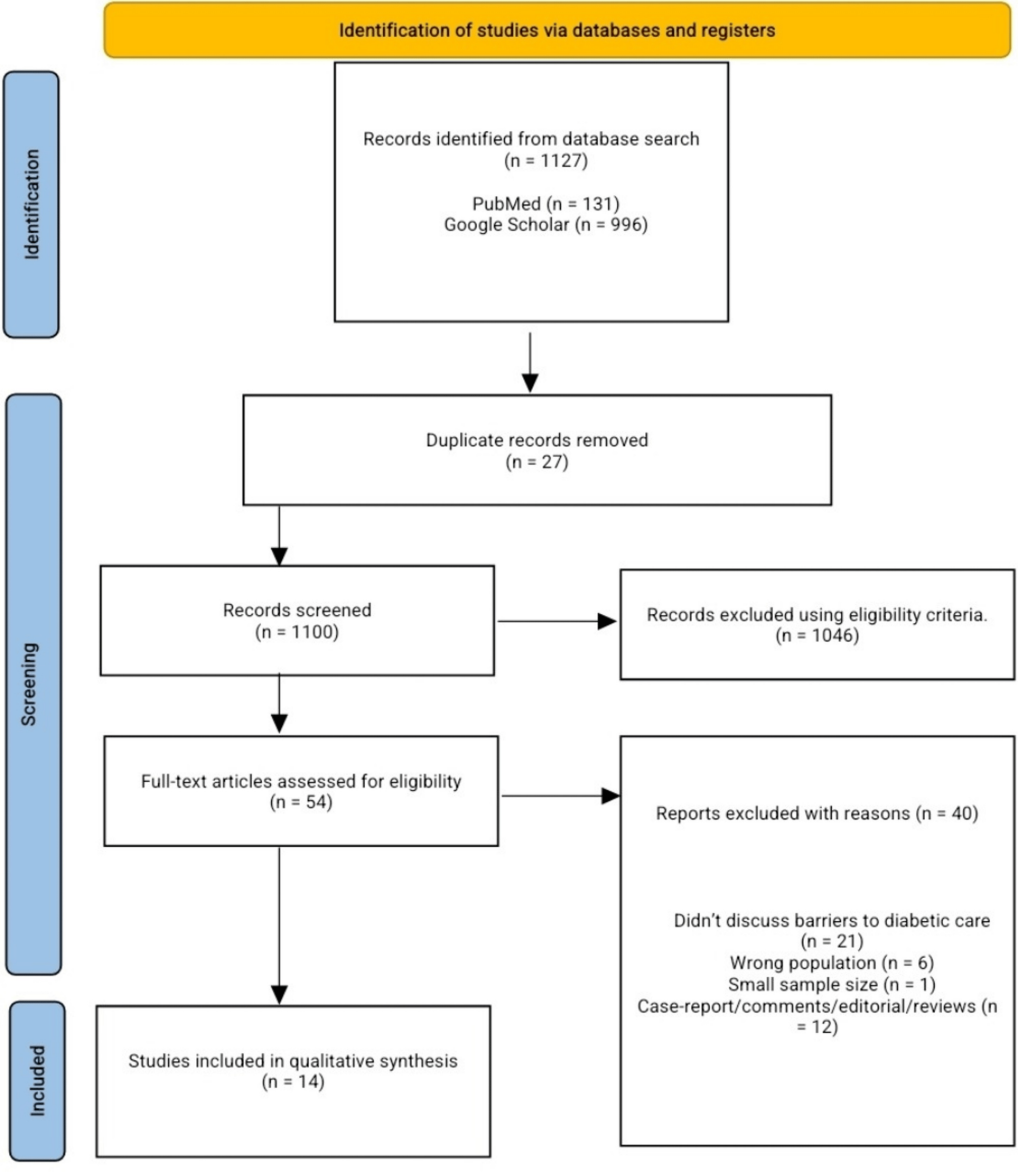
PRISMA flow chart

Our search across databases yielded 1,127 articles; 27 duplicates were removed, 1,100 articles were screened for title and abstract, and 1,046 articles were excluded following our predefined eligibility criteria. Fifty-four articles underwent full-text screening for possible inclusion in the final qualitative analysis and data extraction; 14 articles were included for analysis. We excluded 21 because they didn't discuss barriers/difficulties in diabetes care, 6 were the wrong population (developed country, co-existence of other chronic diseases), 1 article had a sample size of less than 10, and 12 articles were reviews, comments, case reports, or editorials. The PRISMA flow chart is displayed in Figure 1.

Study Characteristics

A total of 1,843 individuals participated in this study, including individuals with diabetes mellitus and healthcare providers, who were represented across 14 included studies conducted in 12 developing countries. India and Ethiopia contributed the highest number of studies (two each).

The majority of participants were patients with diabetes, with reported gender distribution indicating 621 males and 1,019 females; some studies did not report gender-specific data. The mean age of participants varied widely across studies, reflecting diverse populations. 

The duration of diabetes among participants ranged from a minimum of six months to a maximum of nine years. Study periods spanned from 2005 to 2025, indicating both historical and recent perspectives on barriers to diabetes care. The included studies comprised a mix of populations, including diabetes patients, healthcare practitioners, and caregivers with varying study designs and sample sizes. The characteristics of the included studies are summarized in Table 1.

**Table 1:** Study characteristics

Author	Country	Year	Sample size	Gender	Mean age	population	Diabetes duration
Ugwu et al. [9]	Nigeria	2020	129	M: 80 F: 49	N/A	Diabetes specialists, endocrinology trainees, and other healthcare professionals	N/A
Vanderlee et al. [10]	Bangladesh	2015	220	M: 86 F: 134	43	Diabetic patient	5
Chona et al. [11]	Tanzania	2024	35	M: 19 F: 16	58	Diabetic patient	N/A
Haque et al. [12]	South Africa	2005	140	N/A	N/A	Medical officer	N/A
Halali et al. [13]	Iran	2016	146	M: 24 F: 122	53	Diabetic patient	6
Joseph et al. [14]	India	2017	110	M: 58 F: 52	26	Diabetic patient	8
Al-Azri et al. [15]	Oman	2011	19	M: 11 F: 8	41	Diabetic patient	N/A
Yimer et al. [16]	Ethiopia	2025	47	N/A	N/A	Health care practitioner, Diabetic patient	N/A
Taha et al. [17]	Egypt	2011	80	M: 23 F:57	51	Diabetic patient	9
Alfian et al. [18]	Indonesia	2025	455	M: 144 F: 311	N/A	Diabetic patient	N/A
Reshid et al. [19]	Ethiopia	2022	27	M: 10 F: 17	N/A	Healthcare worker, Diabetic patient	N/A
Rohillaa et al. [20]	India	2024	28	M: 9 F: 19	35	Diabetic parents	N/A
Kiçaj et al. [21]	Albania	2025	30	M: 14 F: 16	56	Diabetic parents	6 months
Birabwa et al. [22]	Uganda	2019	377	M: 143 F: 234	49	Diabetic parents	4

Barriers to Diabetes Care

Barriers to diabetes care identified across the included studies were categorized into four major domains: patient level, physician level, healthcare system level, and socio-economic factors.

Patient-level barrier: Financial constraints were the most frequently reported barrier across all included studies [9-11,16,17,19-21], significantly affecting access to medication, diagnostic services, and follow-up care. Other commonly reported patient-level barriers include: low diabetes knowledge and awareness [9,16], poor medication adherence (forgetting medications) [10,12,18], cultural and religious beliefs influencing treatment choices [9,19], psychological burden, including stress and emotional distress [20,21], fear of hypoglycemia and injectable therapies [10,12,18,19], difficulty adhering to dietary recommendations and lifestyle modifications [10,13,14,16,17,21], and lack of family or social support and stigma [16,19,21]. Additional factors, such as advanced age, comorbidities, and low physical activity level, further complicate disease management [10,16,17,21].

Physician-level barrier: Several studies highlighted deficiencies at the provider level, particularly inadequate knowledge or training in diabetes management [9,12,16,19,22], poor communication skills and language barriers [12,13,15], and limited capacity for patient education and counseling. These factors contributed to suboptimal patient understanding and adherence to treatment plans.

Healthcare system-level barrier: Health system challenges were prominent across studies and include shortage of trained healthcare professionals (diabetologists, nurses, dieticians) [9,11,12,16,19,22], poorly equipped healthcare facilities [9,16,22], limited availability of essential medications and glucometers [11,12,16,19,22], high workload among healthcare staff [19,22], fragmented care including lack of continuity with the same provider [12,19], long waiting times and inefficient appointment systems, and limited access to diagnostic services [12,15,19].

Socioeconomic and structural barriers: Broader systemic barriers include limited health insurance coverage, high out-of-pocket costs, perceived lack of government financial support, and influence of unregulated alternative medicine practices [11]. 

These factors further compounded the challenges faced by patients and healthcare providers in managing diabetes effectively. A detailed categorization of identified barriers is provided in Table 2.

**Table 2:** Barriers to diabetes care

Category	Barrier
Patient level	Financial constraint; low diabetes knowledge/awareness; cultural and religious beliefs; time constraints (being too busy); medication non-adherence; difficulty with glucometer use or insulin dosing; dietary challenges (poor adherence, food dissatisfaction, confusion about dietary recommendations); fear of hypoglycemia; fear of injections; psychological burden (stress, emotional distress); lack of perceived treatment benefit; difficulty resisting food temptation; advanced age and comorbidities
Physician-level	Poor knowledge of diabetes care; communication barriers (including language); lack of motivation and confidence; limited patient counseling
Healthsystem level	Shortage of healthcare professionals (including diabetologists and dietitians); poor access to healthcare facilities; dysfunctional primary healthcare systems; scarcity of medications and glucometers; high workload; technological challenges; lack of continuity of care; long waiting times and poor appointment systems; limited access to diagnostic services; inadequate health education and counseling resources; lack of educational materials; unregulated alternative medicine practices
Socioeconomic level	Limited health insurance coverage; lack of government financial support; food insecurity and limited access to appropriate diet

Discussion

This review highlights the complex and interconnected nature of barriers to diabetic care in developing countries. This systematic review synthesized evidence from 14 studies conducted across 12 developing countries to identify barriers impeding effective diabetes care. The findings reveal a multifaceted challenge encompassing patient-level, provider-level, health system-related, and socio-political barriers. Consistent with previous studies, financial hardship emerged as the most pervasive obstacle, underscoring the structural inequities in healthcare financing across low- and middle-income countries (LMICs) [23-25].

Patient-Level Barriers

Financial constraints were the most commonly cited patient-level barrier, reflecting both the direct costs of medication and the indirect costs of accessing care. This aligns with findings from Kibirige et al. [26] in sub-Saharan Africa and Syed et al. [27] in the Middle East, which highlight out-of-pocket expenditure as a major impediment to adherence. The absence of universal health coverage in many LMICs often forces patients to choose between medication and essential living costs [25].

Low diabetes knowledge and awareness were recurrently reported, consistent with the findings of Zowgar et al. [28] and more recent evidence by Stephani et al. [29], emphasizing limited literacy and health education as determinants of poor self-care. Misconceptions about diabetes, reliance on traditional or religious healing, and cultural beliefs further contribute to treatment discontinuity, as shown in studies from Ethiopia and Nigeria [9,16].

Psychological barriers, including emotional distress, stigma, and denial, were also notable. Similar to Barakat et al. [30], this review observed that fear of hypoglycemia and anxiety regarding insulin injections led to poor adherence and glycemic control. The lack of social or familial support, particularly among women and elderly patients, exacerbates these challenges [21,22].

Physician and Provider-Level Barriers

Inadequate provider knowledge of diabetes management and limited training were frequently reported, mirroring findings from Mogre et al. [31], Maneze et al. [32], and Pastakia et al. [23]. Many physicians in resource-limited settings lack continuing professional development opportunities, resulting in suboptimal clinical decisions and inconsistent care practices. Moreover, communication barriers between physicians and patients, particularly in multilingual or low-literacy contexts, impede effective counseling [13,15].

The shortage of trained healthcare professionals, especially diabetologists and dietitians, remains a persistent issue. According to Besancom et al. [33] and Mathew et al. [34], health systems in developing countries suffer from maldistribution of healthcare workers, particularly in rural areas, leading to long waiting times and limited continuity of care. These gaps undermine early diagnosis and long-term management.

Health System Barriers

At the system level, this study identified poor infrastructure, limited diagnostic capacity, and scarcity of essential medicines and glucometers as recurring barriers. These findings echo Adeline [35] and El Klatman et al. [36], who reported that most clinics in LMICs experience frequent stockouts of insulin and diagnostic tools. The overburdened healthcare workforce and inefficient appointment systems further constrain service delivery [19].

Rapid staff turnover and lack of continuity of care were also evident, aligning with Maneze et al. [32], who noted that inconsistent provider relationships reduce patient engagement and adherence. Weak primary healthcare (PHC) systems, insufficient referral structures, and fragmented service delivery models compound these problems [24].

Socioeconomic Barriers

The broader socio-political context, limited government funding, weak health insurance coverage, and poor governance remain critical determinants of diabetes care disparities. Similar to WHO [37] and Manne-Goehler et al. [25], our findings emphasize that insufficient political commitment to non-communicable disease (NCD) control in LMICs perpetuates a cycle of underinvestment and inequity.

Limited health insurance coverage, reported in several included studies [10,17,18], often results in catastrophic expenditures, particularly in rural populations. Additionally, access to healthy food options and safe spaces for physical activity remains constrained by socioeconomic conditions and urbanization patterns [31,37,38].

When compared to previous global reviews, such as Pastakia et al. [23] and Abdul et al. [24], the present study underscores that while awareness of diabetes and its complications is rising, systemic challenges in financing, workforce distribution, and infrastructure persist. Interventions must therefore be multi-tiered, addressing not only patient education but also strengthening health systems through policy reform, sustainable financing, and workforce training.

Digital health innovations, including mobile health (mHealth) programs, have shown potential to overcome communication and access barriers in many countries [39]. However, the implementation of such technologies must consider literacy levels, cost barriers, and cultural appropriateness.

Strengths and Limitations

A major strength of this systematic review lies in its comprehensive synthesis of multi-level barriers to diabetes care across 12 developing countries, offering both breadth and contextual depth. By integrating data from studies conducted between 2005 and 2025, this review captures the evolution of diabetes care challenges over two decades, highlighting persistent systemic inequities as well as emerging issues related to technological change and healthcare financing. The inclusion of perspectives from patients, healthcare providers, and policy contexts provides a holistic understanding of diabetes care ecosystems, moving beyond patient-centric barriers to encompass physician and health system determinants.

Another strength is the application of rigorous eligibility criteria aligned with PRISMA guidelines, ensuring methodological transparency and replicability. Only studies that explicitly addressed barriers to diabetes care in developing countries were included, enhancing thematic relevance and specificity. Furthermore, the review draws from a diversity of geographical and socio-economic contexts, spanning sub-Saharan Africa, South Asia, the Middle East, and Southeast Asia, which strengthens the external validity of the findings and enhances their applicability to a broad range of low- and middle-income settings (LMICs).

This review also highlights the interconnectedness of structural and behavioral barriers, integrating patient-level challenges, such as financial hardship and poor health literacy, with systemic constraints like workforce shortages, limited diagnostic capacity, and weak primary healthcare infrastructures. The triangulation of these findings with existing global evidence underscores its contribution to global health discourse by contextualizing diabetes care disparities within the broader framework of non-communicable disease (NCD) management and universal health coverage (UHC) goals.

However, several limitations warrant acknowledgment. First, heterogeneity among the included studies in terms of design, methodology, and participant characteristics limits direct comparability. Most included studies were qualitative or cross-sectional, potentially restricting causal inference regarding the identified barriers. While qualitative insights are valuable for depth, the lack of quantitative data constrains the ability to estimate the relative magnitude or statistical significance of each barrier. Our search was limited to PubMed and Google Scholar, excluding other databases such as Scopus, Web of Science, Embase, and CENTRAL. This may result in the omission of studies.

Second, there is a degree of geographical imbalance in the evidence base. Countries such as India, Ethiopia, and Nigeria are overrepresented, while regions like Latin America, the Pacific Islands, and parts of North Africa remain underexplored. This imbalance may reflect publication bias, resource availability for research, or differences in data accessibility.

Third, although publication bias was minimized through broad database searching, grey literature and non-English studies were excluded, which may have led to the omission of relevant findings, particularly from Francophone and Lusophone African countries. The reliance on published data may also introduce a reporting bias, as studies with significant or policy-relevant findings are more likely to be published.

Additionally, contextual variations in health system structures, including differences in insurance models, decentralization, and cultural determinants, mean that the barriers identified may not uniformly apply across all developing nations. The findings should therefore be interpreted as indicative patterns rather than universally generalizable conclusions.

Finally, while the review provides a unique categorization of barriers into patient, physician, healthcare system, and structural domains, data on interventional outcomes were limited. Few studies assessed the effectiveness of strategies aimed at overcoming these barriers, highlighting a critical evidence gap in implementation science. Future research should therefore focus on evaluating scalable, context-sensitive interventions, such as community-based health worker programs, telemedicine, and integrated chronic disease management models, particularly in rural and low-resource settings.

## Conclusions

This review demonstrates that barriers to diabetes care in developing countries are multifactorial, with financial constraints and health system limitations emerging as the most critical drivers of poor outcomes. Patient-level challenges, such as low health literacy and medication non-adherence, are compounded by shortages of trained healthcare personnel, limited diagnostic capacity, and a fragmented care delivery system. These factors contribute to delayed diagnosis, suboptimal treatment, and increased morbidity. Addressing these limitations requires coordinated, system-level interventions, as policymakers must prioritize sustainable healthcare financing, strengthen workforce capacity, and improve access to essential medicines and diagnostic services. Integrating diabetes care into the primary healthcare system and expanding health education initiatives will be essential to achieve effective diabetes management in resource-limited settings.

## Appendices

Appendix A 

**Table 3:** Search strategy

Database	Search query	outcome
PubMed	(((barrier) AND (Diabetes)) AND (management)) AND (developing country)	131
Google Scholar	"barrier" "Diabetes" "management" "developing country"	996

Appendix B 

**Table 4:** JBI cross-sectional critical appraisal tool

Checklist question	Ugwu et al [8].	Vanderlee et al [9].	Chona et al [10].	Haque et al [11].	Halali et al [12].	Joseph et al [13].	Al-Azri et al [14].	Yimer et al [15].	Taha et al [16].	Alfian et al [17]	Reshid et al [18]	Rohillaa et al [19]	Kiçaj et al [20]	Birabwa et al [21]
Were the criteria for inclusion in the sample clearly defined?	Y	Y	Y	Y	Y	Y	Y	Y	Y	Y	Y	Y	Y	Y
Were the study subjects and the setting described in detail?	Y	Y	Y	Y	Y	Y	Y	Y	Y	Y	Y	Y	Y	Y
Was the exposure measured in a valid and reliable way?	Y	Y	Y	Y	Y	Y	Y	Y	Y	Y	Y	Y	Y	Y
Were objective, standard criteria used for measurement of the condition?	Y	Y	Y	Y	Y	Y	Y	Y	Y	Y	Y	Y	Y	Y
Were confounding factors identified?	Y	Y	Y	Y	Y	Y	Y	Y	Y	Y	Y	Y	Y	Y
Were strategies to deal with confounding factors stated?	Y	Y	Y	Y	Y	Y	Y	Y	Y	Y	Y	Y	Y	Y
Were the outcomes measured in a valid and reliable way?	Y	Y	Y	Y	Y	Y	Y	Y	Y	Y	Y	Y	Y	Y
Was appropriate statistical analysis used?	Y	Y	Y	Y	Y	Y	Y	Y	Y	Y	Y	Y	Y	Y
